# Mammalian Otolin: A Multimeric Glycoprotein Specific to the Inner Ear that Interacts with Otoconial Matrix Protein Otoconin-90 and Cerebellin-1

**DOI:** 10.1371/journal.pone.0012765

**Published:** 2010-09-15

**Authors:** Michael R. Deans, Jonathan M. Peterson, G. William Wong

**Affiliations:** 1 Department of Otolaryngology, Head and Neck Surgery, Johns Hopkins University School of Medicine, Baltimore, Maryland, United States of America; 2 Center for Hearing and Balance, Johns Hopkins University School of Medicine, Baltimore, Maryland, United States of America; 3 Center for Sensory Biology, Johns Hopkins University School of Medicine, Baltimore, Maryland, United States of America; 4 Department of Physiology, Johns Hopkins University School of Medicine, Baltimore, Maryland, United States of America; CNRS, France

## Abstract

**Background:**

The mammalian otoconial membrane is a dense extracellular matrix containing bio-mineralized otoconia. This structure provides the mechanical stimulus necessary for hair cells of the vestibular maculae to respond to linear accelerations and gravity. In teleosts, Otolin is required for the proper anchoring of otolith crystals to the sensory maculae. Otoconia detachment and subsequent entrapment in the semicircular canals can result in benign paroxysmal positional vertigo (BPPV), a common form of vertigo for which the molecular basis is unknown. Several cDNAs encoding protein components of the mammalian otoconia and otoconial membrane have recently been identified, and mutations in these genes result in abnormal otoconia formation and balance deficits.

**Principal Findings:**

Here we describe the cloning and characterization of mammalian Otolin, a protein constituent of otoconia and the otoconial membrane. Otolin is a secreted glycoprotein of ∼70 kDa, with a C-terminal globular domain that is homologous to the immune complement C1q, and contains extensive posttranslational modifications including hydroxylated prolines and glycosylated lysines. Like all C1q/TNF family members, Otolin multimerizes into higher order oligomeric complexes. The expression of *otolin* mRNA is restricted to the inner ear, and immunohistochemical analysis identified Otolin protein in support cells of the vestibular maculae and semi-circular canal cristae. Additionally, Otolin forms protein complexes with Cerebellin-1 and Otoconin-90, two protein constituents of the otoconia, when expressed *in vitro.* Otolin was also found in subsets of support cells and non-sensory cells of the cochlea, suggesting that Otolin is also a component of the tectorial membrane.

**Conclusion:**

Given the importance of Otolin in lower organisms, the molecular cloning and biochemical characterization of the mammalian Otolin protein may lead to a better understanding of otoconial development and vestibular dysfunction.

## Introduction

The mammalian inner ear is a remarkable sensory structure consisting of the vestibule, which detects motion and facilitates vestibular function, and the cochlea, which is dedicated to hearing. The ability to maintain equilibrium and proper orientation in space is critical for the survival of motile organisms, and the vestibular system is highly conserved throughout evolution [1], [2]. The vestibule comprises five separate sensory organs, each containing hair cell receptors. The utricle and saccule are responsible for sensing linear acceleration, and the three semi-circular canal cristae detect head rotation. In each sensory organ the hair cells are covered by an acellular gel matrix or membrane. The otoconial membrane covers hair cells in the maculae of the utricle and saccule, and the cupula surrounds hair cells in each of the cristae. Movements of these membranes in response to motion deflect the stereocilia bundles on the underlying hair cells, opening mechanosensitive channels and leading to the generation of vestibular-evoked potentials [3].

Recently, several protein constituents of the acellular gel matrix have been identified in mammals, including α- and β-tectorin [4], [5], otogelin [6], [7], and otoancorin [8]. In the mouse, α- and β-tectorin are components of the otoconial membrane and the tectorial membrane that contacts auditory hair cells in the cochlea, but are absent from the cupula [5]. Otogelin is present throughout all of the acellular gels [6], while otoancorin is specifically located at the interface between the sensory epithelia and the overlying gel. Thus, otoancorin functions to anchor the gel matrix to the underlying sensory epithelia [8]. The expression of these genes is also highly restricted to the inner ear, and mutations in these genes result in pronounced hearing and/or balance deficits [8], [9], [10], [11], [12], [13].

A unique feature of the otoconial membrane that is absent from the tectorial membrane or the cupula is the presence of biomineralized CaCO_3_ crystals called otoliths, or ear stones, in teleost fish and otoconia, or ear dust, in mammals [14], [15], [16]. The mammalian otoconial membrane holds thousands of otoconia (∼10 µM), and the entire complex is anchored to the hair cell kinocilia. Teleost fish, by comparison, lack an otoconial membrane; nonetheless, three large otoliths still appear tethered to the underlying hair cells [17], [18], [19]. The critical function of otoconia and otoliths is to impart inertial movements in response to gravity or linear acceleration, which stimulates the underlying sensory hair cells by deflecting their stereocilia bundles [3]. Consistent with this, all mouse mutants lacking otoconia (such as *tilted*, *head slant*, and *head tilt* mice) have severe balance deficits [20], [21], [22], [23].

Otoconia and otoliths contain an inner core matrix made up of glycoproteins (termed Otoconins) and proteoglycans, while the outer surface consists mostly of precipitated CaCO_3_ [15]. These CaCO_3_ crystals exist in three major polymorphs: calcite (found in mammals and birds), aragonite (found in amphibians and fish), and vaterite (found in primitive jawfish such as garfish) [24]. It is generally believed that the different CaCO_3_ polymorphs in otoconia and otoliths are determined by the major matrix proteins, which bind calcium and make up the organic core [25]. In mammals and birds, the major core protein is a highly glycosylated glycoprotein named Otoconin 90 (Oc90/95) [26], [27]; in amphibians, it is Otoconin 22 [28]; in primitive jawfish, it is Otoconin 54 [25]; and in teleost fish, it is otolith matrix protein (Omp) [17], [18]. Targeted deletion of Oc90 in mice results in balance deficits due to absent or abnormal (few and large) otoconia [29], [30]. Similarly, morpholino knockdown of Oc90 orthologs in fish lead to an aberrant otolith phenotype [31].

How otoconia are formed and subsequently embedded in the otoconial membrane during inner ear development remains unclear. In mammals, this process is initiated during embryogenesis and is completed during early postnatal maturation [32], [33]. In teleost fish, development of the otolith also initiates early in otic development, however otoliths continue to grow throughout the life of the fish [2]. The source of calcium in the endolymph is thought to be provided by the extrusion of Ca^2+^ [via the plasma membrane Ca^2+^-ATPase isoform 2 (PMCA2)] from the hair cells [34]. Consistent with this, PMCA2-null mice exhibit severe balance deficits resulting from the complete absence of otoconia [34]. During otoconia formation, CaCO_3_ is precipitated about the otoconial core matrix proteins. Each matrix protein is expressed in different regions of the utricular and saccular sensory epithelium, resulting in a corresponding variation in otoconia size and shape.

One of the most common forms of vestibular dysfunction and vertigo in humans is benign paroxysmal positional vertigo (BPPV) [35], [36], a condition in which otoconia dislodged from the utricle become trapped in the semi-circular canals. Approximately 50% of dizziness or vertigo in the elderly population is attributed to BPPV [37], representing a major risk factor for falls, bone fractures, and accidental death [38]. Although BPPV can be caused by head trauma, inner ear infection, ototoxic drugs, or age-related degeneration of otoconia, the etiology of the majority of BPPV cases is unknown. Important underlying factors are the processes that anchor and embed otoconia within the otoconial membrane; these likely involve specific interactions between otoconial proteins and the matrix proteins of the acellular gel.

We have recently identified and characterized a family of secreted glycoproteins belonging to the C1q/TNF family of proteins [39], [40], [41]. One novel C1q/TNF family member is homologous to teleost Otolin, an inner ear-specific, collagenous protein important for the growth and function of otolith structures of the vestibular system [17], [18], [19]. Morpholino knockdown of *otolin* transcripts in zebrafish demonstrates that Otolin is required for the proper anchoring of otoliths onto the sensory epithelium and for the overall stability of the otolith matrix [17]. Expression of a mammalian ortholog of Otolin has been reported in mouse inner ear [30], however *otolin* cDNA has not been cloned and Otolin protein has not been characterized biochemically.

In the present study we show that *otolin* encodes a secreted multimeric glycoprotein with extensive posttranslational modifications. Expression of *otolin* is highly restricted to the inner ear, and Otolin protein contributes to all extracellular matrices contacting sensory hair cells, including the otoconial membrane and the tectorial membrane of the cochlea. Further, Otolin can physically interact with otoconia proteins such as Oc90 [26], [27] and Cerebellin (Cbln1) [42], suggesting that Otolin is also a component of protein complexes involved in otoconia formation. Together, this evidence suggests that Otolin is an important component of the extracellular matrices of the inner ear and is necessary for auditory and vestibular function.

## Materials and Methods

### Identification and cloning of mouse otolin

Multiple C1q/TNF family member cDNAs and protein sequences were used to query the NCBI GenBank databases and identify several mouse expressed sequence tags (ESTs) that encode a novel protein with significant homology to the globular C1q domain of chum salmon (*Oncorhynchus keta*) Otolin (GenBank accession number BAB84561). Based on EST clones and genomic sequences corresponding to mouse *otolin*, a nested PCR approach was used to clone the entire coding region from 17-day mouse embryo cDNA (Clontech). Primers 5′-CAGTGCTGTCCAGGAGAAGGATTGG-3′ and 5′-ATAGGAATAGTTGACACTATGCTGG-3′ were used in first round PCR (35 cycles) using a high-fidelity Pfu DNA polymerase (Stratagene). An aliquot (3 µl) of this reaction was used as template for a second round of amplifications (35 cycles) using primers 5′-CACCCATAAGCCTCGAATATGTGG-3′ and 5′-TAGAATAAATCAGAA GTACAGTGTCC-3′. The resulting PCR product was purified and cloned into the pCRII TOPO cloning vector (Invitrogen). The entire cDNA insert was sequenced and results were deposited into GenBank with the accession number DQ002405.

### cDNA constructs

The C-terminal FLAG (DYKDDDDK peptide) and HA- (YPYDVPDYA peptide) tagged Otolin were generated by PCR and cloned into the pCRII TOPO vector (Invitrogen). Tagged cDNAs were excised from pCRII TOPO using EcoRI enzyme (New England Biolabs) and cloned into the mammalian expression vector, pCDNA3.1 (Invitrogen). Mouse Oc90 cDNA was cloned from a mouse embryo (Day-17) cDNA pool (Clontech) using primers 5′-CCTACACCTTGTCCTCTGCACTGC -3′ and 5′-ACTGAGGGCCAAAGGGCT CAGACAG -3′. A total of 36 rounds of PCR amplification were carried out using a high fidelity *Pfu* polymerase (Stratagene) in the presence of 7% DMSO. Mouse Oc90 protein exists in multiple isoforms due to alternative splicing [27], and the cDNA we cloned corresponds to the described version B that lacks the peptide segment “AGEVRADTLTTLSRTK” between the two phospholipase A2 domains [27]. Cbln1 and Cbln4 cDNA clones were obtained from Open Biosystems. The C-terminal HA-tagged Oc90, Cbln1, and Cbln4 constructs were generated by the same method as described for epitope-tagged Otolin. All constructs were verified by DNA sequencing. The mammalian expression vectors encoding C-terminal HA-tagged Adiponectin, CTRP1, CTRP2, CTRP3, CTRP5, CTRP6, CTRP9, and CTRP10 used in this study were described in our previous studies [39], [40], [41].

### Quantitative real time PCR analysis

Quantitative real time PCR was used to screen a mouse multiple tissue cDNA panel (Clontech, Mountain View, CA, USA) and RNAs isolated from the inner ear of P0 mouse for the presence of *otolin* transcripts. The following q-PCR primers were used in this study: *Otolin* forward, 5′-AAGGGCTTAAAGCCGTCCAGTGG-3′; *Otolin* reverse, 5′-GTGTCCAGAGAGAAGCTCTCG-3′; *Oc90* (NM_010953)forward, 5′- GCTCAGTCTGGCATCAACTCC-3′; *Oc90* reverse, 5′- CTGGTATCTGTGGGCTTTTCAG-3′; *Cbln1* (NM_019626) forward, 5′-CAGGAGGGGAGTGAGAAAGAG -3′; *Cbln1* reverse, 5′-GGGAGTGTGCAGAGCTAAGC-3′; *Cbln2* (NM_172633) forward, 5′-GAGCCCATCGTG CTAGAGG -3′; *Cbln2* reverse, 5′-CCTGAGCGCACAGAAATGC-3′; *Cbln3* (NM_019820) forward, 5′-GGGACGGAATGGCACAAAC -3′; *Cbln3* reverse, 5′-CACTCCCCCTCCAGT AGGAC -3′; *Cbln4* (NM_175631) forward, 5′- GCCGTTCTGCTGATTCTAGTG -3′; *Cbln4* reverse, 5′-CTGGGTTCGAGTCGCACAC-3′; *Sparc* (NM_009242) forward, 5′-GTGGAAAT GGGAGAATTTGAGGA -3′; *Sparc* reverse, 5′-CTCACACACCTTGCCATG TTT-3′; *SparcL1* (NM_010097) forward, 5′-GGCAATCCCGACAAGTACAAG-3′; *SparcL1* reverse, 5′-TGG TTTTCTATGTCTGCTGTAGC-3′; *Chondromodulin-1* (NM_010701)forward, 5′- CCTGAGGA CGTTGAGTTTTGC -3′; *Chondromodulin* reverse, 5′-CAGCTCCTACCTTGAGCAGC-3′; *Otopetrin* (NM_172709) forward, 5′- GAAGGGCTGGGTTGCCTTAG -3′; *Otopetrin* reverse, 5′- ACACGTTCAGTCCATACTG GC-3′; *Collagen II α1* (Col2a; BC052326) forward, 5′- CAGGATGCCCGAAAATTAGGG-3′; *Col2a* reverse, 5′- ACCACGATCACCT CTGGGT-3′; *otoancorin* (NM_139310) forward, 5′- TGGAGGTGCCTATCAGAGAGA-3′; *otoancorin* reverse, 5′- GTGAGATCCAGTAACGCATTCA-3′; otogelin (NM_013624) forward, 5′-CGTT GCCAGTTGGTGTATAATGT-3′; *otogelin* reverse, 5′- GAAAAGTAGTAGTACAGGCC GTC-3′; *α-tectorin* (NM_009347) forward, 5′- TTCGCTCTTGTTCGGCACC-3′; *α-tectorin* reverse, 5′- CTCAGAGGAGCTTCCGTCATC-3′; *β-tectorin* (NM_009348) forward, 5′- GTCA GGGCCTTCGTTTTGCT-3′; *β-tectorin* reverse, 5′- CTCGGGGATTTTAGTGATGATGG-3′; *Stereocilin* (NM_080459) forward, 5′- CTCAGTCTTTGGATGCTGGTC-3′; *Stereocilin* reverse, 5′- GGAACGCAGAGAACCGTGA-3′; *18S RNA* forward, 5′-GCAATTATTCC CCATGAACG-3′; *18S RNA* reverse, 5′-GGCCTCACTAAACCATCCAA-3′. The default PCR protocol was used on an Applied Biosystems Prism 7000 Sequence Detection System. Mouse inner ear cDNAs were synthesized from 1 µg of total RNA and 200 ng of random hexamers using the Superscript II RNase H-Reverse Transcriptase (Invitrogen). For quantitative PCR, samples were analyzed in 25-µl reactions (10 ng of cDNA, 900 nmol of primer, 12.5 µl of master-mix, and water) according to the standard protocol provided in SyBR® Green PCR Master Mix protocol (Applied Biosystems).

### Generation of Otolin-specific antibody

The C-terminal FLAG-tagged Otolin was produced and purified from the supernatants of transiently transfected HEK293T cells. Briefly, 24 h after transfection, DMEM media containing 10% FBS were replaced by serum-free Opti-MEM I media supplemented with vitamin C (0.1 mg/mL). Supernatants were collected 3 times, every 48 h, pooled and purified using the anti-FLAG affinity gel (Sigma), and eluted with 150 µg/mL of FLAG peptide (Sigma). Purified proteins were dialyzed against 20 mM HEPES buffer (pH 8.0) containing 135 mM NaCl in a 10 kDa cut-off Slide-A-Lyzer dialysis cassette (Pierce). Rabbit polyclonal antibody directed at purified recombinant Otolin was produced by immunizing NZW rabbits as described previously [39]. Sera were collected and tested for their ability to recognize HA-tagged Otolin by Western blot analysis.

### HEK293T Cell Transfection

HEK293T were cultured in DMEM containing 10% fetal calf serum supplemented with 2 mM L-glutamine, 100 units/mL penicillin, and 100 µg/mL streptomycin. Transient transfections were performed in HEK293T cells using lipofectamine 2000 reagent (Invitrogen). Twenty-four hours after transfection, cells were washed and cultured in serum-free Opti-MEM I medium (Invitrogen) supplemented with vitamin C (0.1 mg/mL) for 24–48 h before the conditioned media was collected for Western blot analysis using the anti-FLAG M2 (Sigma) or anti-HA (clone 3F10 - Roche) monoclonal antibody. A sample of the supernatant from Otolin transfectant was incubated with PNGaseF (New England Biolabs), chondroitinase ABC, or *O*-glycosidase (Sigma) to determine the presence of N-linked glycans, chondroitin sulfate proteoglycans, or *O*-linked glycans, respectively.

### Western blotting

Supernantants (25 µl) from transfected cells were suspended in 60 µl NuPAGE LDS sample buffer (Invitrogen) containing reducing agent (β-mercaptoethanol), heated at 90°C for 10 min, and separated on 10% NuPAGE Bis-Tris gel (Invitrogen). Proteins from gels were transferred to 0.2 µm Protran BA83 nitrocellulose membrane (Whatman), blocked in 5% non-fat milk for 1 h, and probed overnight with the mouse anti-FLAG M2 (1∶5000), rat anti-HA (1∶2000) monoclonal antibody, or polyclonal rabbit anti-Otolin antibody (1∶5000) in the presence of 5% non-fat milk. Immunoblots were washed 3× (5–10 min each) in PBS containing 0.1% Tween 20 and incubated with the sheep anti-mouse-HRP or the goat anti-rat-HRP (Amersham Biosciences) (1∶5000) for 1 h. Blots were washed 3× (10 min each) in PBS containing 0.1% Tween 20, developed in ECL reagent (Millipore) for 2–5 min, and exposed to Blue XB-1 film (Kodak).

### Co-immunoprecipitation Analysis

An aliquot of supernantants (250–350 µl) collected from transfected cells was combined with 500 µl of IP buffer (150 mM Tris-HCL, pH 7.4, 150 mM NaCl, 1 mM EDTA, and 1% Triton X-100) and subjected to immunoprecipitation using the anti-FLAG M2 affinity gel (Sigma) or rabbit anti-Otolin antibody in the presence or absence of 5 mM EDTA. Samples were rotated for 4 h or overnight at 4°C, washed 4 times with IP buffer, resuspended in SDS-PAGE loading buffer containing β-mercaptoethanol, and subjected to Western blot analysis. For native gel electrophoresis, immunoprecipitates were eluted with either FLAG peptide (150 µg/ml) or 0.1 M glycine buffer (pH 3.5) and immediately resuspended in 2× Novex Native TrisGly sample buffer (Invitrogen), followed by non-reducing, non-denaturing, native gel electrophoresis.

### Reducing and non-reducing gel electrophoresis

Protein samples (recombinant Otolin and ground-up P0 mouse inner ear) were suspended in NuPAGE LDS sample buffer (Invitrogen) in the presence or absence of reducing agent (β-mercaptoethanol), heated at 90°C for 10 min, and separated on 4–12% NuPAGE Bis-Tris gels in NuPAGE MOPS SDS running buffer at 195 volts for 4 hr. Separated proteins were transferred onto 0.2 µm Protran BA83 nitrocellulose membrane (Whatman) and subjected to Western Blot analysis.

### Native Gel Electrophoresis

Protein samples (recombinant Otolin, immunoprecipitates, and P0 mouse inner ear) in Novex Native TrisGly sample buffer (Invitrogen) were separated on 4% Novex Tris-Glycine gels (Invitrogen) in Novex Tris-Glycine native running buffer (Invitrogen) at 125 volts for 4 hr, transferred onto PVDF membrane (Bio-Rad) in Novex Tris-Glycine transfer buffer (Invitrogen), and subjected to immunoblot analysis using the anti-FLAG or anti-Otolin antibody. NativeMark protein standard (Invitrogen) was used in native gel electrophoresis to estimate the apparent molecular weight of native Otolin. In the case of P0 mouse inner ear, the excised tissues were rapidly frozen in liquid nitrogen and ground to powder. The powder was resuspended in 2× Novex Native TrisGly sample buffer (Invitrogen) prior to separation in 4% Novex Tris-Glycine gel. Because Otolin is tightly associated with otolith, the very mild method we used only extracted a very small percentage of the total native Otolin.

### Glycoprotein detection

Approximately 50 ng of purified recombinant FLAG-tagged Otolin were separated on SDS-PAGE gels, transferred to PVDF membrane, and subjected to ECL glycoprotein detection protocol according to the manufacturer's instructions (GE Health Sciences). Briefly, any carbohydrate moiety on recombinant Otolin was oxidized with sodium metaperiodate and the oxidized sugar aldehyde group was labeled with biotin using biotin-hydrazide [43]. The presence of carbohydrate moiety was then detected using streptavidin conjugated to horseradish peroxidase (HRP) and chemiluminescence substrate (Millipore).

### Gel Filtration Analysis

The supernatant (500 µl) from transfected HEK293T cells, containing FLAG-tagged Otolin, was loaded into an AKTA FPLC and fractionated through a Superdex 200 HR 10/30 column (GE health science) in PBS. The internal diameter of the HR 10/30 column is 10 mm and the height of the packed bed is 30 cm. The total bed volume is 24 ml and the void volume of the column is ∼7.5 ml. In the default setting, the first two fractions (1 ml) were not collected. Aliquots of the collected fractions (0.5 ml each) were subjected to Western blot analysis using the anti-FLAG M2 antibody.

### Mass spectrometry analysis

Purified recombinant Otolin was fractionated on an SDS-PAGE gel, and a single band corresponding to Otolin was excised and subjected to trypsin, chymotrypsin, or AspN digestion. Peptide fragments were then loaded onto the Waters Nano Acquity HPLC coupled to Thermo LTQ linear ion trap mass spectrometer for *ms/ms* analysis. The resulting collision-induced-dissociation spectra were compared against a protein database using SEQUEST (Thermo) to identify the individual peptide and the modified residue as previously described [40].

### Immunofluorescent and Immunohistochemical labeling of inner ear sensory epithelia

Embryonic (E18.5) and early postnatal tissues (P2) from CD-1 mice (Charles River) were prepared for immunofluorescent labeling by immersion fixation in a solution of 4% paraformaldehyde prepared in 67 mM Sorensons' phosphate buffer (pH 7.4) on ice for 2 hours. For immunohistochemistry, P2 ears were dissected to expose the bony labyrinth then immersion fixed using 4% paraformaldehyde and 0.1% glutaraldehyde. After fixation all samples were washed extensively in PBS and cryoprotected by saturation in a series of 10%, 20%, and 30% sucrose prepared in PBS, and then frozen in a block of Neg-50 (Richard Allen Scientific, Kalamazoo, MI) using a dry-ice ethanol bath. Sections were cut from frozen blocks at 20 microns using a Micron Cryostat and collected onto Fisher SuperFrost Plus glass slides.

For immunofluorescent labeling sections were blocked and permeabilized for 30 minutes at room temperature using 5% donkey serum, 1% bovine serum albumin (BSA), and 0.5% Triton X-100 in PBS. Primary antibodies were prepared in blocking solution (5% donkey serum, 1% BSA in PBS) and incubated on the sections overnight at 4°C in a humidified chamber. Slides were washed 4× 10 minutes in PBS, treated with species-specific, Alexa-fluor-conjugated secondaries (Invitrogen, Carlsbad, CA) prepared in blocking solution, and incubated for 2 hours at room temperature. Slides were washed again and mounted using Biomeda Gelmount. For immunohistochemical detection P2 tissue was sectioned, blocked, and labeled overnight with the Otolin antibody, followed by detection using Vectashield ABC Elite labeling kit (Vector, Burlingame, CA) per manufacturer's recommendations, then histochemical detection using ImmPACT DAB substrate (Vector).

For whole mount labeling of E18.5 organ of Corti, tissue was fixed using 4% paraformaldehyde as described and cochleas were dissected to remove the tectorial membrane and expose the sensory epithelia. Samples were blocked and permeabilized as described and incubated with primary antibodies diluted in 5% donkey serum, 1% BSA, and 0.1% Tween20 prepared in PBS at 4°C overnight. Samples were washed 4× 30 minutes with PBS supplemented with 0.05% Tween 20, followed by incubation with species-specific Alexa Fluor-conjugated secondary antibodies (Invitrogen) for 3 hours at room temperature. Tissue was washed again, mounted on slides using Biomeda GelMount, and imaged by confocal microscopy using a Zeiss LSM 510 confocal microscope. The following antibodies and reagents were used in this study: rabbit anti-Otolin (this study), goat anti-Calretinin (Millipore, Bedford, MA), donkey anti-goat AlexaFluor488, donkey anti-rabbit AlexaFluor594, phalloidin AlexaFluor488 (Invitrogen, Carlsbad, CA).

## Results

**Figure 1:**
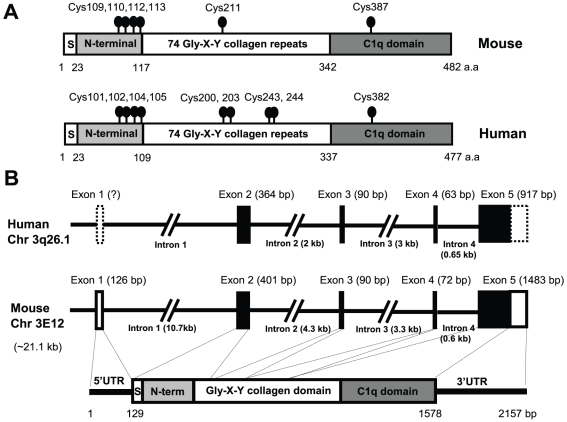
Cloning of the mouse *otolin* cDNA. *A*, The deduced mouse and human otolin proteins consist of four domains— a signal peptide (S), an N-terminal region with four Cys residues, a collagen domain with 75 Gly-X-Y repeats, and a globular C1q domain. All Cys residues with their amino acid positions are indicated with a *ball-and-stick*. Note that human otolin has three more Cys residues located in the collagen domain compared to mouse otolin. *B*, The exon/intron structures of human and mouse *otolin* gene. Dashed boxes indicate 5′ and 3′UTR that cannot be determined due to lack of homology between the human and mouse gene. The size of each exon and intron are indicated. The region of the cDNA encoded by each exon is indicated by dash line.

The mouse ortholog of *otolin* was identified based upon expressed sequence tags (ESTs) and mouse genomic sequences in the NCBI GenBank database with significant homology to the chum salmon (*Oncorhynchus keta*) *otolin* gene (accession number BAB84561). The mouse *otolin* gene is ∼21 Kb in size, located on chromosome 3E12, and consists of 5 exons and 4 introns (Fig. 1B). Comparisons of mouse and human genomic sequences revealed that the exon/intron structure of *otolin* is conserved between these species (Fig. 1B). A nested PCR approach was used to clone the entire *otolin* coding region (∼1.5 Kb) from embryonic day 17.5 (E17.5) mouse embryo cDNAs (Fig. S1). The mouse *otolin* mRNA is 2157 bp in size, and consists of a 129 bp 5′UTR, a 1449 bp coding region, and 579 bp of 3′UTR sequences. The deduced Otolin protein is 482 amino acids long, with a signal peptide, four conserved Cysteines in the N-terminus at positions 109, 110, 112, and 113, a collagen domain with 74 Gly-X-Y repeats, and a C-terminal globular domain that is homologous to the immune complement C1q (Fig. 1A). Therefore, Otolin is a new member of the expanding C1q/TNF family of proteins [44], [45].

**Figure 2:**
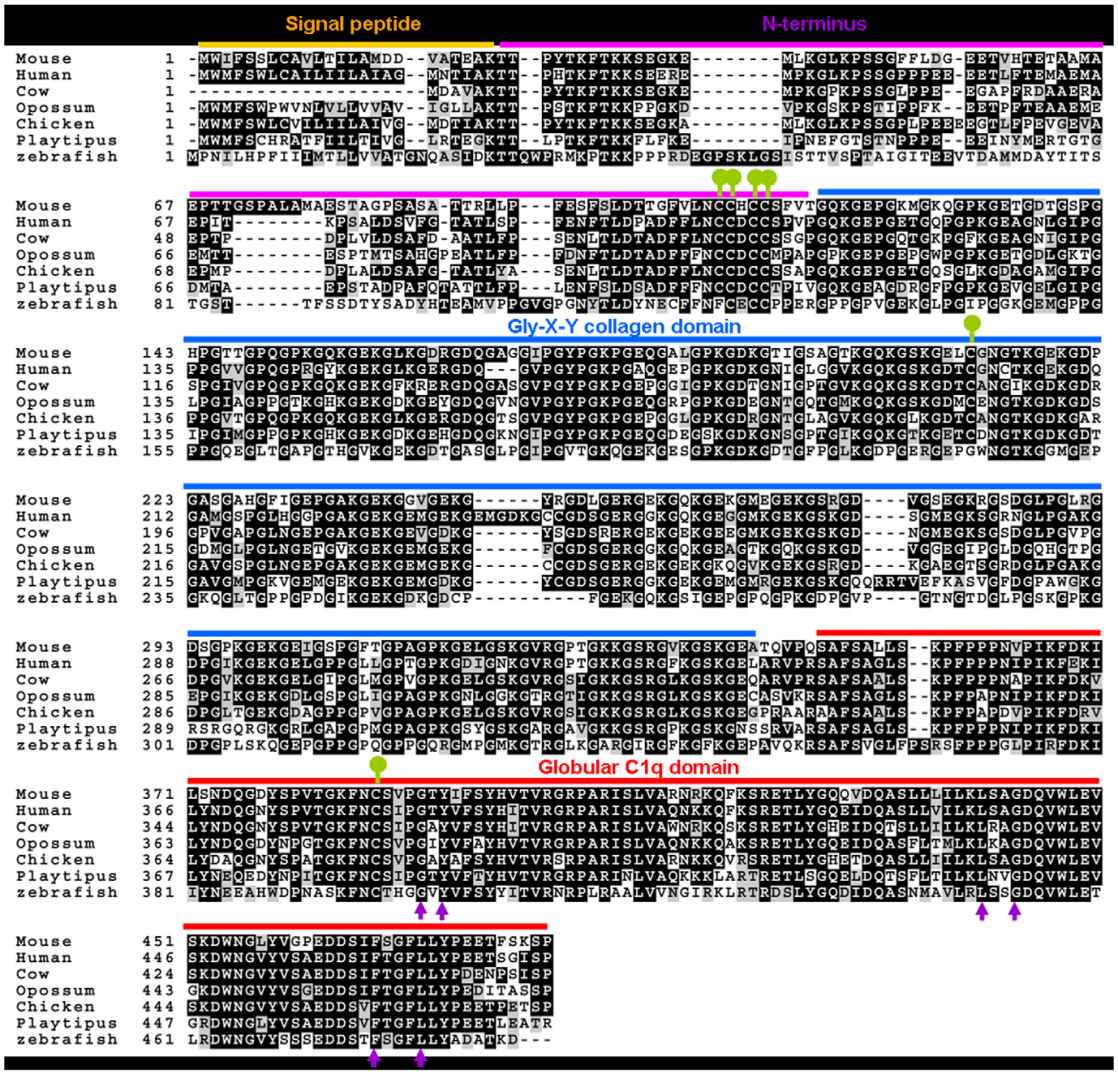
Alignment of otolin sequences from multiple species. ClustalW alignment of Otolin protein sequences extracted from the draft genome sequences of human (*Homo sapiens*; accession number NP_001073909), mouse (*Mus musculus*; DQ002405), cow (*Bos taurus*; XP_603387.3), opossum (*Monodelphis domestica*; XP_001369147), chicken (*Gallus gallus*; XP_426716.2), platypus (*Ornithorhynchus anatinus*; XP_001512453), and zebrafish (*Danio rerio*; NP_001093211). Identical amino acids are *shaded* and gaps are indicated by a dash line. All the conserved Cys residues are indicated by a green ball-and-stick. Signal peptide (yellow line), N-terminus (purple line), collagen domain (blue line), and globular C1q domain (red line) are indicated. Conserved residues found in all C1q/TNF family members [46] are indicated by arrows.

The primary sequence of Otolin is highly conserved throughout evolution (Fig. 2 and Fig. S3 and Table S1). This is particularly evident in the C-terminal globular domain, with 86, 83, 87, 81, 79, 79, 75, 77, and 53% amino acid identity between mouse and its corresponding counterparts in human, dog, cat, cow, opossum, horse, platypus, chicken, and zebrafish, respectively. This region is thought to be a significant functional domain for interactions with other proteins and receptors. In addition, structure based alignments of Adiponectin, complement C1q, and TNF family members (TNF-α, TNF-β, and CD40L) revealed four highly conserved residues (Gly-159, Tyr-161, Phe-237, and Leu-241 in Adiponectin) that are important in the packing of the protomer's hydrophobic core [46]. These residues are conserved in Otolin (Fig. 2, arrow), as are Cys residues located in the N-terminus (Cys-109, 110, 112, and 113), the collagen domain (Cys-211), and the C-terminal globular domain (Cys-387) (Fig. 2, *ball-and-stick*). Of all the C1q-domain containing proteins, mouse Otolin shares the highest degree of amino acid identity (52%) in the globular domain with fish sacullar collagen [47], a protein found only in the inner ear of fish (Fig. S2).

**Figure 3:**
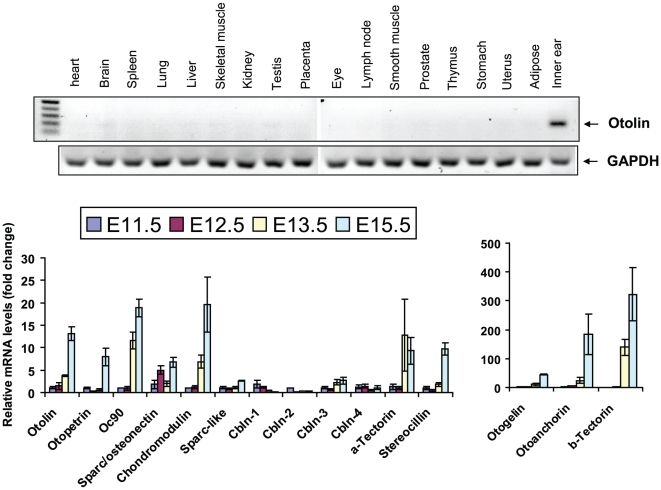
Expression of otolin transcripts in mouse tissues. *A*, The expression profile of otolin mRNA in various mouse tissues as revealed by a semi-quantitative PCR. *B*, Real time PCR analysis of the expression profiles of otolin and other known inner ear genes during development. All expression data are normalized to 18S RNA.

A semi-quantitative PCR analysis revealed that mouse *otolin* expression is restricted to the inner ear (Fig. 3A). A 40-cycle semi-quantitative PCR reaction failed to detect *otolin* transcripts from seventeen major adult mouse tissues (heart, brain, spleen, lung, liver, muscle, kidney, testis, placenta, eye, lymph node, smooth muscle, prostate, thymus, stomach, uterus, adipose tissue). Consistent with these real-time PCR results, a survey of *otolin* EST distributions in GenBank indicated that *otolin* transcript is present only in the inner ear (data not shown). Further, the time course of *otolin* expression during inner ear development was similar to genes encoding otoconial membrane constituents, including *Oc90*, *otogelin*, *otoancorin*, *α-tectorin*, *β-tectorin*, and *sparc* (Fig. 3B).

**Figure 4:**
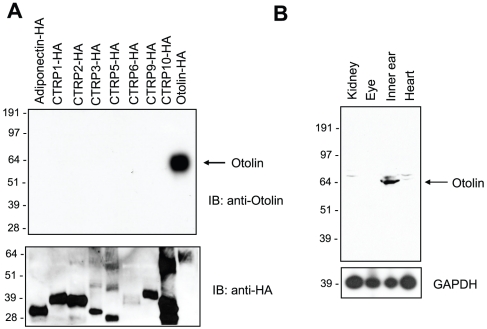
Specificity of Otolin Antibody. *A*, Western blot analysis of the HA-tagged adiponectin, CTRP1, CTRP2, CTRP3, CTRP5, CTRP6, CTRP9, CTRP10, and otolin proteins using an anti-otolin (top panel) or an anti-HA antibody (bottom panel). *B*, Western blot analysis of mouse kidney, eye, inner ear, and heart tissue lysates using the anti-otolin antibody.

**Figure 5:**
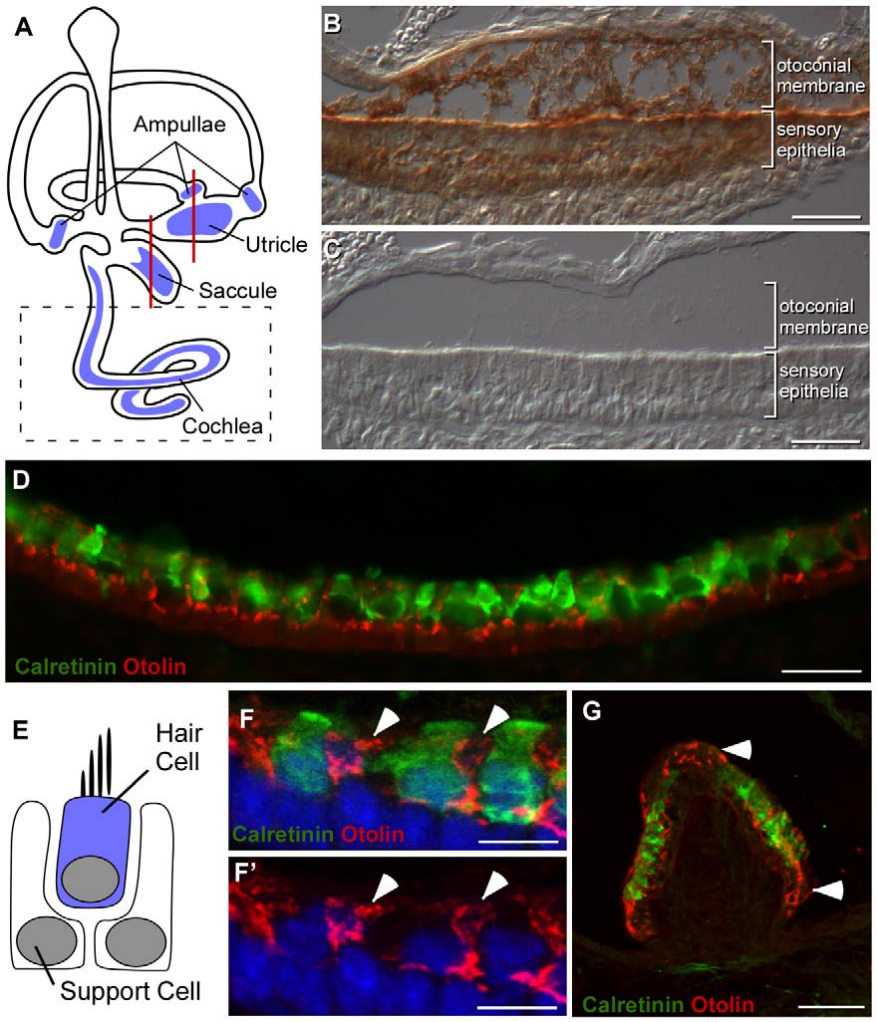
Distribution of Otolin protein within the vestibular sensory epithelia of the inner ear. *A,* Hair cell receptors are located in one of six sensory epithelia as indicated by blue shading in this illustration of the mouse inner ear. Vestibular hair cells are found in the maculae of the utricle and saccule and the cristae of the semi-circular canal ampullae. Auditory hair cells are found exclusively in the cochlea. Red lines indicate the relative position of the tissue sections illustrated in the remaining panels. Dashed box indicates the auditory region described in greater detail in Fig. 6*B*, Histochemical detection of Otolin protein in P2 utricle. Otolin is present in the extracellular matrix of the otoconial membrane and within cells of the sensory epithelia. *C,* No signal is present in control experiments lacking Otolin antisera. *D,* Immunofluorescent labeling of Otolin (red) and the hair cell marker Calretinin (green) reveals little colocalization in the P2 saccule, indicating that Otolin is synthesized by support cells. Note that the otoconial membrane is not preserved during this labeling protocol. *E,* Vestibular hair cells and support cells form a pseudostratified epithelium, with the support cell nuclei located below the hair cells and support cell processes surrounding individual hair cells. *F&F*', High power confocal image demonstrating Otolin immunofluorescence (red, arrowheads) in support cell processes located between neighboring hair cells (green, F). *G,* In the semi-circular canal cristae, Otolin is expressed in support cells surrounding the hair cells (green) and located adjacent to the hair cell regions (arrowheads). Scale bars: 50 µm B,C,G; 25 µm D, 10 µm F,F'.

An Otolin-specific antibody was generated and used to determine the distribution of Otolin within the inner ear and to identify the cells producing this predicted extracellular glycoprotein. This antibody does not cross-react with other members of the C1q/TNF family (Fig. 4A) and labels a single ∼65 kD protein in postnatal day 4 (P4) mouse inner ear lysate (Fig. 4B). The contribution of Otolin protein to the composition of the otoconial membrane was determined by preparing P2 inner ear tissue using fixation protocols optimized to preserve these extracellular matrices (see methods). Immunohistochemical detection revealed Otolin protein throughout the otoconial membrane located above the utricle (Fig. 5B,C). Labeling was also present in the sensory epithelia, indicating that cells in this region synthesize and secrete Otolin into the extracellular matrix. To determine whether Otolin protein is produced by hair cells or support cells, we immunofluorescently labeled the vestibular maculae from P2 mouse for Otolin and the hair cell marker Calretinin. Lighter fixation protocols that are compatible with immunofluorescence, but are not sufficient to preserve the otoconial membrane, were used for these experiments. We found no overlap between Otolin and Calretinin immunofluorescent signals, indicating Otolin production by support cells and not vestibular hair cells (Fig. 5D). Additionally, in the vestibular maculae, hair cells and support cells formed pseudostratified epithelia with hair cells positioned above the support cell soma and apical support cell processes extending between neighboring hair cells to contact the lumenal surface (Fig. 5E). When viewed at higher magnification using confocal microscopy, Otolin immunofluorescence was detected in these support cell processes, but not in calretinin-labeled hair cells (Fig. 5F,F'). A similar distribution of Otolin protein in support cells and not hair cells occurred in cristae, the sensory epithelia housed in the ampullae of the semi-circular canals (Fig. 5G).

**Figure 6:**
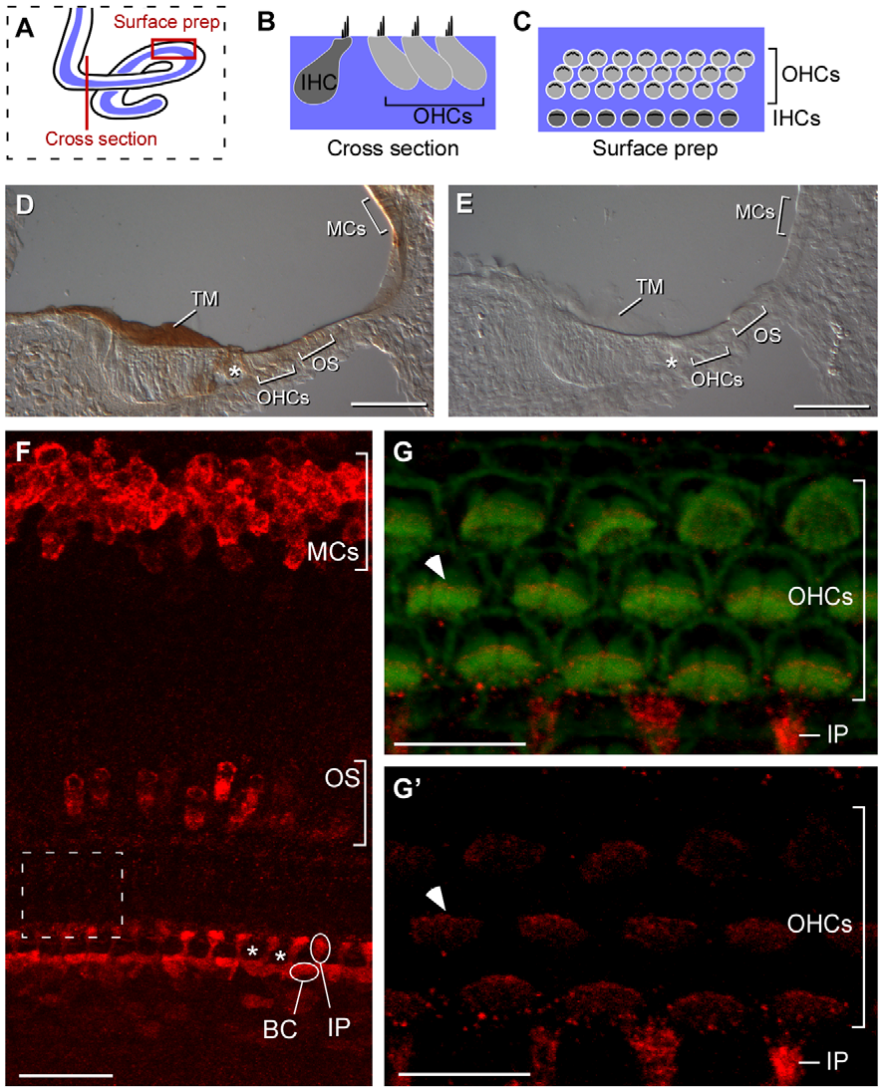
Distribution of Otolin protein in the cochlea. *A–C,* The distribution of Otolin in the auditory system was evaluated in cross sections cut through the cochlea (as indicated by a red line) or whole mount surface preparations (outlined by a red box). (*B*) In cross section the profile of cochlear cell types and overlying tectorial membrane are visible. (*C)* Surface preparations provide a view of the apical surface of cochlear cells without the tectorial membrane. The relative position and organization of cochlear cells in these two views are illustrated using inner hair cells (IHCs) and outer hair cells (OHCs) for reference. (*D*) Histochemical detection of Otolin in neonatal (P2) mouse cross sections reveals Otolin protein throughout the tectorial membrane that contacts auditory hair cells. A subset of cochlear support cells is labeled surrounding the IHCs (asterisk) in addition to marginal cells and the outer sulcus. (*E),* No signal is present in control experiments lacking Otolin antisera. *(F)* In surface preparations prepared from E18.5 mouse, Otolin (red) can be visualized in inner phalangeal cells (IP) and border cells (BC), two classes of support cells located adjacent to the IHCs. The positions of individual examples are indicated. Although the IHCs are not labeled in this image the position of two IHCs are marked by asterisks. Additional non-sensory cells producing Otolin that contribute to formation of the tectorial membrane can also be visualized in cochlear whole mounts. This includes marginal cells of the stria vascularis and a small population of cells in the outer sulcus. (*G),* High magnification confocal imaging of OHC stereocilia labeled with phalloidin (green) also shows Otolin protein (red) at the tips of the stereocilia bundle (one example marked by arrowhead) resulting from contact between the stereocilia and the Otolin-rich tectorial membrane. (*G*') Otolin immunofluorescence detected in the red channel from panel G. The region being imaged is indicated by the dashed box in *F*. *TM* (Tectorial Membrane), *MCs* (Marginal Cells), *IHC* (Inner Hair Cell), *OHC* (Outer Hair Cell), *IP* (Inter Phalangeal Cell), *BC* (Border Cell), *OS* (Outer Sulcus). Scale bars: 100 µm *D,E*; 20 µm *F*, 10 µm *G,G*'.

Using histochemical detection methods, we also found Otolin reactivity in the tectorial membrane, an extracellular matrix that contacts auditory hair cells of the cochlea (Fig. 6D,E). The tectorial membrane was easily visualized in cross-sections cut through the cochlea, as indicated in Figure 6A. To identify the source of Otolin production in the cochlea, we evaluated Otolin distribution by confocal imaging of surface preparations of cochleas dissected from E18.5 embryos. In these preparations the tectorial membrane was removed during dissection to facilitate imaging of the hair cell stereocilia. The position and orientation of this ‘*en face*’ view relative to cochlear cross sections is illustrated schematically in Figures 6A–C. At this stage, Otolin immunofluorescence labeled two populations of support cells that are adjacent to the inner hair cells (IHCs); these are the interphalangeal cells and the border cells (Fig. 6F). Otolin was also present in non-sensory epithelia cells of the cochlea, including the marginal cells of the stria vascularis and a small population of cells distributed throughout the outer sulcus. These are likely a subset of Claudius cells (Fig. 6F). It is interesting to note that at higher magnification Otolin was also detected at the tips of the outer hair cell (OHC) stereocilia (Fig. 6G, G', arrowheads). Because Otolin protein cannot be detected in OHC soma (Fig. 6F), this fluorescence is likely an artifact resulting from natural contacts that occur *in vivo* between the stereocilia and the tectorial membrane. In summary, using histochemical and immunofluorescent labeling, we found that Otolin is not restricted to the vestibular apparatus of the inner ear. Instead, Otolin protein is present in all extracellular matrices contacting hair cell stereocilia.

**Figure 7:**
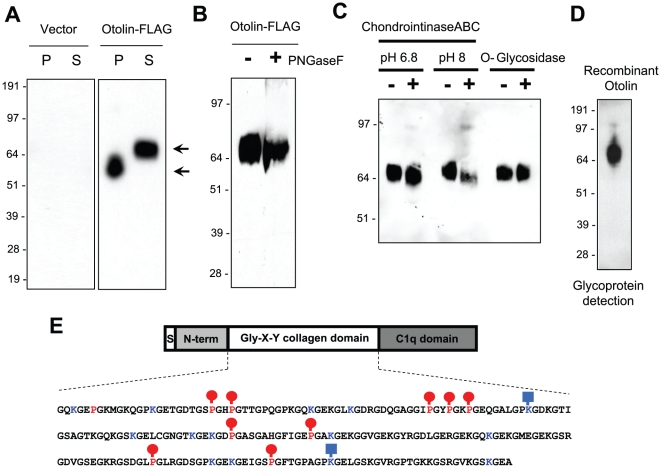
Otolin is a secreted glycoprotein with extensive posttranslational modifications. *A*, Western blot analysis of the cell pellets (P) and supernatant (S) from transfected HEK293T cells using the anti-FLAG antibody. *B*, Recombinant otolin-FLAG was incubated with (+) or without (−) peptide, N-glycosidase F (PNGaseF), to determine the presence of N-linked glycans. Proteins were immunoblotted with the anti-FLAG antibody. *C*, Recombinant otolin-FLAG was incubated with (+) or without (−) chondroitinase ABC or *O*-glycosidase to determine the presence of chondroitin sulfate proteoglycans and *O*-linked glycans, respectively. Chondroitinase ABC preferentially digests hyaluronic acid at pH 6.8 and chondroitin sulfate at pH 8. Proteins were immunoblotted with the anti-FLAG antibody. *D*, A metaperiodate oxidation-based method was used to detect the presence of carbohydrate moieties on recombinant otolin (See *Materials and Methods*). *E*, Mass spectrometry analyses of recombinant otolin. All lysine residues that lie within the consensus sequence [GX**K**G(E/D)] are highlighted in blue, and those that are glycosylated are indicated with a square-and-stick. All proline residues (in the Gly-X-Pro context) that lie within the collagen domain are highlighted in red, and those that are hydroxylated are indicated with a ball-and-stick.

The biochemical characteristics of Otolin were determined by expression in mammalian HEK293T cells that secrete recombinant Otolin (Fig. 7A), consistent with this protein having a signal peptide. We observed Otolin isoforms of different apparent molecular weights in the cell pellet fraction versus the conditioned medium suggesting that the mature, secreted Otolin contains posttranslational modifications. Otolin contains two potential *N*-linked glycosylation sites (Asn-213 and Asn-386). However, when treated with *N*-glycanase (PNGase F), no shift in the apparent molecular weight of secreted Otolin was observed on immunoblot, indicating the absence of *N*-linked glycans (Fig. 7B). An *in silico* search of putative *O*-glycosylation sites (http://www.cbs.dtu.dk/services/NetOGlyc/) [48] in the mouse Otolin protein predicted ten putative residues (Ser-72 and Thr-60, 62, 69, 70, 81, 135, 138, 146, and 147) that can be potentially modified with the attachment of *O*-linked glycans. To determine if Otolin is posttranslationally modified with the attachment of proteoglycans or *O*-linked glycans, recombinant Otolin was enzymatically treated to remove proteolgycans (chondroitinase ABC) or *O*-linked glycans (*O*-glycosidase). Neither treatment changed the apparent molecular weight of Otolin on immunoblot (Fig. 7C), indicating the absence of chondroitin sulfate proteoglycans, hyaluronic acids, and *O*-linked glycans. However, recombinant Otolin clearly contains carbohydrate moieties, as revealed by the metaperiodate oxidation-based glycoprotein detection method (Fig. 7D). Proteins with collagen domains (e.g., Collagen) contain posttranslational modifications that enhance the stability of their triple helical collagen structure [49]. Typically, proline on the third position of the Gly-X-Pro repeat is hydroxylated [49], and lysine within the consensus GX**K**G(E/D) is hydroxylated and glycosylated with α-1,2-glucosyl-galactosyl disaccharide moieties [40], [50], [51]. Mouse Otolin contains 10 proline residues (Pro-123, 141, 144, 174, 177, 180, 222, 234, 288, and 306) that can potentially be hydroxylated and 14 lysine residues (Lys-120, 132, 156, 162, 189, 207, 216, 219, 237, 261, 297, 300, 315, and 339) that can potentially be hydroxylated and glycosylated (Fig. 7E). The modification state of each of these residues was analyzed by mass spectrometry, and nine out of the ten proline residues were found to be hydroxylated (Fig. 7E)- the lone exception was Pro-123. Within the collagen domain, two of the fourteen lysine residues (Lys-189 and Lys-315) were hydroxylated and glycosylated with a glucosyl-galactosyl group. The remaining lysine containing peptides were too small to be detected on the mass spectrometer, and their posttranslational modification status remains to be determined.

**Figure 8:**
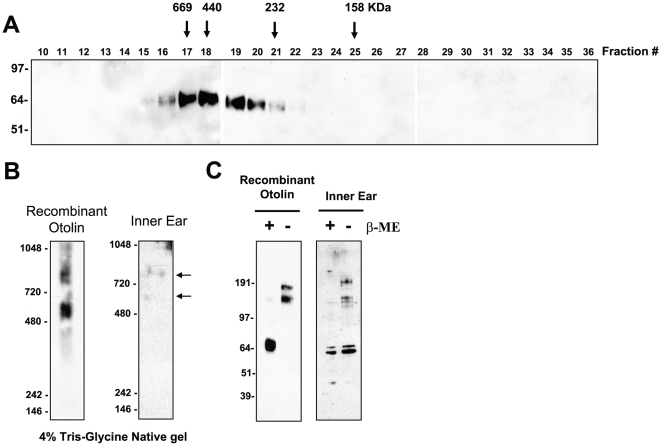
Otolin assembles into higher order multimeric complexes. *A*, Supernatant containing FLAG-tagged otolin was loaded onto superdex 200 FPLC column and 0.5 ml fractions were collected. Fractions 10 to 27 were analyzed by immunoblot analysis using the anti-FLAG antibody. Arrows with the molecular weight markers of 669, 440, 232, and 158 kDa correspond to the peak elution fraction of molecular standard thyroglobulin, ferritin, catalase, and aldolase, respectively. *B*, 4% Tris-Glycine native gel immunoblot analysis [see ***MATERIALS AND METHODS***] of purified recombinant otolin and endogenous otolin (indicated by arrows) present in the P0 mouse inner ear, detected with anti-otolin antibody. *C*, Recombinant otolin and endogenous otolin present in the P0 mouse inner ear were subjected to reducing (+) and non-reducing (−) SDS-PAGE and Western blot analysis using the anti-otolin antibody. B-ME, β-mercaptoethanol.

All members of the C1q/TNF family of proteins form a trimer as their basic structural unit [44]. Some of the trimers are further assembled into higher order structures corresponding to the hexameric and HMW oligomeric forms [41], [52], [53], [54]. Gel filtration analysis revealed that Otolin also forms higher order multimeric complexes (Fig. 8A). Although we presumed that Otolin forms trimers and possibly higher order structures similar to other C1q/TNF family members, due to the low resolution of the Superdex 200 HR 10/30 column, we cannot distinguish different oligomeric structures of Otolin. Additionally, all the proteins used to calibrate the FPLC column consist of spherical/globular proteins. In contrast, Otolin has a rigid triple helical collagen domain consisting of 75 Gly-X-Y repeats; hence, Otolin has a much larger Stoke's radius compared to the globular molecular standards. Consequently, Otolin oligomers eluted from the gel filtration column with an apparent molecular size much greater than the globular protein standards of the same molecular weight. In an orthogonal approach, we employed a non-reducing, non-denaturing native gel immunoblot technique to confirm that recombinant Otolin, and endogenous Otolin from P0 mouse inner ear, form higher order multimeric complexes (Fig. 8B). It appears that both recombinant and endogenous otolin exist in two distinct complexes with different sizes. The diffuse band of recombinant Otolin on native gel is due to variable degrees of glycosylation (Fig. 7). In the presence of reducing agent, the higher order multimeric complexes of both recombinant and endogenous otolin collapsed to a single or doublet band around ∼65 KDa on an SDS-PAGE immunoblot (Fig. 8C). In the absence of reducing agent, both recombinant and endogenous otolin migrated as 160 and 190 kDa bands, indicating that they have similar disulfide linkages (Fig. 8C).

**Figure 9:**
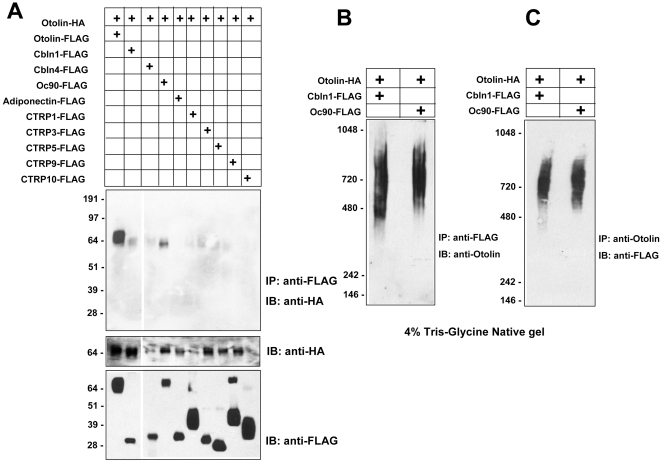
Otolin forms physical complexes with Cbln1 and Oc90. *A*, Supernatants from transfected HEK293T cells co-expressing HA-tagged otolin with different FLAG-tagged proteins were subjected to immunoprecipitation (IP) with the anti-FLAG affinity gel followed by immunoblot (IB) analysis using the anti-HA antibody (top panel). Middle and bottom immunoblot panels indicate the presence of HA- or FLAG-tagged proteins in the input media. *B*, Otolin co-immunoprecipitated (IP) with FLAG-tagged Cbln1 and Oc90 were analyzed by 4% Tris-Glycine native gel immunoblot (IB) analysis. *C*, Cbln-1 and Oc90 co-immunoprecipitated (IP) with otolin were analyzed by 4% Tris-Glycine native gel immunoblot (IB) analysis. IP, immunoprecipitation; IB, immunoblot.

Otoconin-90 (Oc90), Sparc/Osteonectin, and Cerebellin (Cbln) are recently identified protein constituents of mammalian otoconia [26], [27], [42]. Cerebellins also belong to the C1q/TNF family of proteins similar to Otolin [44]. Human and mouse have four Cerebellins (Cbln1–4), which are all secreted proteins [55], and Cbln3 requires Cbln1 for secretion [56]. Our observation of Otolin protein in the otoconial membrane of P2 mouse suggests that this group of molecules may interact during formation of the otoconial complex. To determine if Otolin physically interacts with these proteins, epitope-tagged Oc90 and Cbln-1 were co-expressed with Otolin in HEK293T cells, and the secreted proteins were subjected to co-immunoprecipitations (Fig. 9A). These analyses revealed that Otolin can form physical complexes with Oc90 and Cbln-1 but not with other related C1q-containing proteins (Fig. 9A). Further, we observed weak interactions of otolin with Cbln-4 and CTRP3, indicating that otolin may interact with these two proteins as well. Because variations were observed in the co-expressions of epitope-tagged proteins, and due to the inability to mimic *in vitro* the native condition (i.e., the inner ear) in which these proteins may physically associate, we cannot rule out the possibility that otolin may interact with Cbln-4 and/or CTRP3 *in vivo*. The presence of 5 mM EDTA did not affect the ability of Otolin to interact with Cbln-1 or Oc90, suggesting that the physical interactions do not require calcium (data not shown). Additionally, using Tris-Glycine native gel immunoblot analysis, we show that Cbln-1 and Oc90 interact with the oligomeric form of Otolin (Fig. 9B–C).

## Discussion

When expressed in mammalian cells, secreted Otolin forms higher order multimeric structures. As revealed by reducing and non-reducing SDS-PAGE and native gel immunoblots, both recombinant and endogenous otolin have similar disulfide linkages. The formation of multimeric complexes likely underlies interactions between Otolin and other glycoproteins and proteoglycans to regulate the process of otoconia growth and adherence to the otoconial membrane, as well as formation of the otoconial and tectorial membranes. Two major types of posttranslational modifications of the collagen domain that are known to be important for protein structure, stability, and function are proline hydroxylation [49] and lysine glycosylation [57], [58]. Using mass spectrometry, we demonstrated that nine out of the ten proline residues (within the sequence Gly-X-Pro) of the mouse Otolin protein are hydroxylated, consistent with similar modifications seen in other collagen proteins. Further, all 14 lysine residues [within the sequence GX**K**G(E/D)] in mammalian Otolin proteins are conserved from platypus to humans. Interestingly, only 7 of these 14 lysine residues are conserved in zebrafish. Because lysine glycosylation within the collagen domain has been shown to be important for the proper assembly of HMW oligomeric forms of Adiponectin [58], fish and mammalian Otolin may differ in their oligomeric structures due to differences in the extent of posttranslational modifications. These differences may be reflected in the unique characteristics of otoconial crystals compared to otoliths. Overall, the ability of mammalian Otolin to form higher order multimeric structures that are >600 kDa in size is likely to be important for its role in regulating otoconia seeding and growth, and assembly of the otoconial membrane.

The major core protein of mammalian otoconia is Otoconin-90 (Oc90/Oc95) [26], [27]. Oc90 is expressed at E9.5 in the mouse otocyst, approximately five days before the onset of otoconial mineralization, and provides organic matrix scaffolding for calcium carbonate deposition. Consistent with this, Oc90-null mice have normal hearing but possess balance deficits due to the absence or improper formation of otoconia [29], [30]. Interestingly, in the absence of Oc90, there is a dramatic reduction of Otolin protein in mutant otoconia [30]. This supports our finding that secreted Otolin physically interacts with Oc90 when the two proteins are co-expressed. Thus similar phenotypes might be predicted to occur as a result of mutations or targeted mutagenesis of *otolin.* Likewise, in teleosts Otolin is a major constituent of the otoliths, along with a secreted glycoprotein that is similar to Oc90, called otolith matrix protein (OMP-1). Morpholino knockdown of *otolin* mRNA in zebrafish demonstrates that Otolin is required to anchor the otoliths onto the sensory epithelium and maintain the overall stability of the otolith matrix [17]. Zebrafish Otolin is also synthesized and secreted by a group of non-sensory cells located next to the marginal zone of the sensory epithelium [17]. We similarly found that mouse Otolin is expressed by support cells in the utricle and saccule and is not produced by vestibular hair cells. Together these data are consistent with a general function for Otolin as a large extracellular scaffolding protein that connects the core matrix proteins of the otoconia and otoliths to the acellular gel matrix and sensory epithelia of the inner ear.

One remarkable aspect of otoconia development is that protein components of the otoconia, including Oc90 and Otolin, are expressed throughout the membranous labyrinth of the inner ear- including the cochlea- while otoconia formation is restricted to the utricle and saccule. Thus, it has been postulated that one or more maculae-specific protein(s) interact with Oc90 to catalyze otoconia formation specifically within these compartments [59]. Indeed, it is likely that the same proteins that initiate biomineralization also determine the final crystal structure and size of the otoconia. In one effort to identify such factors, Nagasawa and co-workers cloned a novel otolith matrix protein in Rainbow trout (*Oncorhynchus mykiss*), designated as otolith matrix macromolecule-64 (OMM-64) [60]. OMM-64 is homologous to Starmaker, a zebrafish otolith matrix protein that has been shown to play a role in controlling the shape and size of otoliths [61]. In addition to binding calcium and heparin glycosaminoglycan chains, OMM-64 binds Otolin. Together these complexes form ring-like structures in the otolith matrix that have been suggested to regulate crystal morphology during otolith biomineralization [60]. Although Starmaker is required for normal otolith formation in the zebrafish [61], targeted deletion of its mammalian ortholog, dentin sialoprotein (DSP), in mice revealed no vestibular dysfunction thus far [62], suggesting that the function of Starmaker may be specific to the fish otolith. Additional molecules that may interact with Otolin to direct otoconia development are the C1q/TNF domain-containing Cerebellin proteins. Although the role of Cerebellin (Cbln)-like proteins in fish otolith is not known, we show that mouse Otolin can physically interact with one of the known Cerebellins, Cbln1, when co-expressed. It remains to be determined which Cerebellin isoform is found in mammalian otoconia matrices, but recently Cbln1-null mice were generated [63] and no inner ear defects have been reported.

The size and density of otoconial crystals dictate the extent of hair cell stereocilia bundle deflection and hence the amplitude of the input stimulus to the underlying vestibular hair cells [15]. Consequently, changes in the size and location of otoconial crystals often result in balance deficits [12], [34], [64], [65], [66], [67], [68]. Also significant from a clinical standpoint, otoconial crystals may become dislodged from the acellular gel matrix and become trapped by gravitational pull in the semicircular canals. These conditions are referred to as canalithiasis when the otoconia is misplaced in the canal itself, and cupulothiasis when it is located adjacent to the cristae. Individuals with these conditions suffer from BPPV, the most common cause of vertigo [35], [36]. The molecular underpinning of BPPV is currently unknown; however, Otolin is a significant candidate because its physical association with otoconial matrix proteins and distribution throughout the otoconial matrix suggest that it has an important role in embedding otoconia crystals. Consistent with this is the *otolin* morphant phenotype in zebrafish, in which otoliths are detached from the sensory epithelia. Thus, cloning of the *otolin* cDNA will allow us to assess if mutations in this gene give rise to vestibular dysfunction in patients and is associated with balance deficits such as BPPV.

## Supporting Information

Figure S1 Cloning of the mouse otolin cDNA. A, Based upon ESTs and genomic sequences corresponding to mouse otolin, a nested PCR approach was used to clone the entire coding region. The position of primer pairs 56F1/56R3 and 56F2/56R4 used in the nested PCR are indicated by the arrows. B, The entire coding region (∼1.5 kb) of mouse otolin was amplified from 17-day mouse embryo cDNA (Clontech) using a nested PCR approach.
(0.85 MB EPS)

Figure S2 Phylogentic analysis of otolin. A phylogenetic tree was generated using ClustalW and MEGA program version 4 based on alignment of the globular domain of otolin and other C1q/TNF family members. Percent amino acid identity of each protein to the globular domain of otolin is indicated on the right. GenBank accession number for each of the proteins is: chipmunk (Tamias sibiricus) hibernating protein of 20 kDa (HP-20, BAB68362), HP-25 (BAA02352), HP-27 (AAB20866), C1q-A chain (NP_031598), C1q-B chain (NP_033907), C1q-C chain (NP_031600.2), multimerin-1 (XP_284198), multimerin-2 (NP_878260), emilin-1 (NP_598679), emilin-2 (NP_660140), CTRP1 (NP_064343), CTRP2 (NP_081255), CTRP3 (NP_112150), CTRP4-1 (first globular domain, NP_080437), CTRP4-2 (second globular domain, NP_080437), CTRP5 (AAY21930), CTRP6 (NP_082607), CTRP7 (NP_780634), CTRP9 (DQ002401), CTRP10 (AAY21934), adiponectin/Acrp30 (Q60994), collagen-X (NP_034055), collagen-VIII (NP_031765.2), cerebellin-1 (Cbln-1, NP_062600.2), Cbln-2 (NP_766221), Cbln-3 (NP_062794), Cbln-4 (NP_783439), Lepomis macrochirus saccular collagen (P98085), and otolin (DQ002405). All are mouse proteins except chipmunk HP-20, HP-25, and HP-27, and fish saccular collagen.
(1.11 MB EPS)

Figure S3 Comparison of the domain structures of otolin found in different vertebrate species. The deduced human (Homo sapien), mouse (Mus musculus), opossum (Monodelphis domestica), platypus (Ornithorhynchus anatinus), chicken (Gallus gallus), and fish (Danio rerio) otolin proteins consists of four domains - a signal peptide (S), an N-terminal region with four Cys residues, a collagen domain with 69-74 Gly-X-Y repeats, and a C-terminal globular domain that is homologous to immune complement C1q. All the Cys residues are indicated by ball-and-sticks. Note that different vertebrate species have different numbers of Cys residues in their collagen domain.
(1.30 MB EPS)

Table S1 Amino acid sequence comparison between mouse otolin and its vertebrate orthologs.
(0.03 MB XLS)
